# A New Double-Step Process of Shortening Fibers without Change in Molding Equipment Followed by Electron Beam to Strengthen Short Glass Fiber Reinforced Polyester BMC

**DOI:** 10.3390/ma17092036

**Published:** 2024-04-26

**Authors:** Michael C. Faudree, Yoshitake Nishi

**Affiliations:** 1 Faculty of Liberal Arts and Science, Tokyo City University, Yokohama-shi 224-8551, Japan; 2 Graduate School of Engineering, Tokai University, Hiratsuka-shi 259-1292, Japan; west@tsc.u-tokai.ac.jp

**Keywords:** SGFRP, BMC, impact strength, fiber length, texture, electron beam irradiation

## Abstract

It is vital to maximize the safety of outdoor constructions, airplanes, and space vehicles by protecting against the impact of airborne debris from increasing winds due to climate change, or from bird strikes or micrometeoroids. In a widely-used compression-molded short glass fiber polyester bulk-molded compound (SGFRP-BMC) with 55% wt. CaCO_3_ filler, the center of the mother panel has lower impact strength than the outer sections with solidification texture angles and short glass fiber (SGF) orientations being random from 0 to 90 degrees. Therefore, a new double-step process of: (1) reducing commercial fiber length without change in molding equipment; followed by a (2) 0.86 MGy dose of homogeneous low-voltage electron beam irradiation (HLEBI) to both sides of the finished samples requiring no chemicals or additives, which is shown to increase the Charpy impact value (*a*_uc_) about 50% from 6.26 to 9.59 kJm^−2^ at median-accumulative probability of fracture, *P*_f_ = 0.500. Shortening the SGFs results in higher fiber spacing density, *S*_f_, as the thermal compressive stress site proliferation by action of the CTE difference between the matrix and SGF while the composite cools and shrinks. To boost impact strength further, HLEBI provides additional nano-compressive stresses by generating dangling bonds (DBs) creating repulsive forces while increasing SGF/matrix adhesion. Increased internal cracking apparently occurs, raising the *a*_uc_.

## 1. Introduction

With the increase in frequency and intensity of disaster events from climate change, it is important to advance materials that can withstand the increasingly harsh environmental conditions that can occur. It is crucial to always strengthen materials for maximum safety, with the utmost concern for the environment. Bulk molded compounds (BMCs) have been widely-used across many industries for light-load bearing articles, such as electrical housing, car headlights, and appliance parts. BMCs have advantages over metals in being corrosion resistant and lightweight when used as non-structural parts for aircraft or other vehicles to lower fuel consumption and reduce CO_2_ emissions. Other advantages are their easy formability of complex shaped parts, and their resistance to hot or cold for house appliances and outdoor household articles. BMCs usually have fiber content of ~5 to 30 wt.% [1,2,3,4,5,6,7,8,9,10,11,12] which includes glass [11,12], carbon [13], as well as jute [14] and kenaf [15]. Short glass fiber reinforced polymer–bulk molded compounds (SGFRP-BMCs) are 3-phase fiber-filler-polymer systems constructed with fiber at ~5 to 30 wt.% [1,3,7] and filler of CaCO_3_ at ~35 to 55 wt.% [7,8]. For BMCs in general, fillers of TiO_2_, Al_2_O_3_, SiC, Mg(OH)_2_, ZnO [9], fumed silica [10], fly ash [5], or waste thermosetting BMC [2] have been used. 

Up to now, the combination of shortening fibers and electron beam treatment to enhance mechanical properties of fiber reinforced polymers (FRPs) has not been found in the literature. However, other strengthening methods have always been advancing for FRPs [16,17,18,19,20,21,22,23,24,25]. For example, for long fiber FRP, pre-stressing fibers before intrusion with the polymer melt has been a useful tool to increase mechanical properties [16,17,18,19]. Similar to pre-stressing steel bars in concrete, Pang and Fancey found viscoelastic prestressing of multi-filament nylon 6,6 yarn in a bisphenol-A-based low viscosity epoxy resin increased tensile strength, modulus, and toughness up to 15%, 30%, and 40%, respectively [16]. For a unidirectional glass fiber reinforced polymer (GFRP) epoxy composite, Hadi and Ashton found pre-stressing GFs at 25, 50, 75, 100, and 200 MPa increased tensile strength and elastic modulus [17]. Motahari and Cameron found prestressing fibers increased flexural properties of FRP [18]. Interestingly, Jenkins et al. found by controlling prestressing magnitude and eccentricity, mold-free FRP composites’ internal stress conditions can be manipulated to obtain curved part geometry with high precision [19]. Another method commonly used for strengthening FRPs has been enhancing adhesion at the fiber/matrix interface [20,21,22,23,24,25]. Yuan et al. found that applying good a coupling agent to GFs increased fracture stress, but decreased fracture strain in polyvinyl chloride (PVC) GFRP [20]. Meraghni et al. modelled the effect of interfacial degradation on a short fiber-reinforced polymer containing matrix microcracks [21]. Numerous fiber treatments have been applied to inert CF to increase its weak adhesion to polymer matrix [22,23,24,25], including electrochemical modification [22], electro-polymer coating [23], plasma surface modification [24], and Ni sputtering [25]. 

Past research has shown the mechanical properties of 2-phase fiber/polymer FRP systems that shortening fiber length decreases mechanical properties such as impact strength, tensile stress, and strain [26,27,28,29,30,31,32,33,34], and that longer fibers are desired [35]. For example, in a polypropylene (PP) GFRP at GF wt.% from 3 to 60%, stiffness was found to be lower at shorter fiber lengths below 0.5 mm, and virtually unchanged above 0.5 mm [26]. Above 40 wt.% GF content, modulus was lowered by fiber packing problems and increased in voids [26]. Another study of PP GFRP showed that impact strength was increased as GF length was raised to 6.4 mm, with a strain energy model predicting optimal length to be 8 mm [28]. In a PP carbon fiber reinforced polymer (CFRP), a sequential reduction in mechanical properties, Izod impact, tensile strength and modulus, bending strength and modulus, and Rockwell hardness were found as CF length was reduced from 10 to 5 to 2 to 1 to 0.5 mm [32]. In a CFRP, tensile strength and stiffness were increased by lengthening CF from 2 to 4 mm, but were decreased as CF length was increased further to 6.4 mm. Optimal fiber length was reported to be 4 mm [33]. Numerical modeling has also been carried out on 2-phase GFRP systems showing that as GFs are lengthened, strength increases rapidly at low fiber lengths, especially near the critical length, *l*_c_ of ~1.0 mm, and flattens out at about 5*l*_c_ [29,30]. Fu and Lauke calculated *l*_c_ to be 0.56–0.59 mm for nylon FRP, 1.4 mm for polypropylene (PP) FRP, and 0.68 to 0.84 mm for polybutylene terephthalate (PBI) FRP [29]. For 2-phase systems, below *l*_c_, fibers do not impart stress transfer to the matrix, i.e., they are too short to exhibit shear lag with the matrix during tension. Hence, instead of breakage, the dominant fracture mechanism for fibers below *l*_c_ is pull-out, weakening the 2-phase fiber/polymer system. However, with the 3-phase fiber-filler-polymer system, CTE difference is the dominant mechanism, where shortening fibers to 0.44 mm strengthens, rather than weakens, the composite [36]. For green composites also (2-phase), a trend was found, being that lowering fiber length decreases mechanical strength, such as those with hemp fiber (HF) [34,37], jute fiber (JF) [38,39], sisal fiber (SF) [40], or agave fiber (AF) [41]. For example, in HF-reinforced thermoplastic polyurethane (HFRP), tensile strength was raised from 16 to peak out at 27 MPa by lengthening HFs from 6 mm to 15 mm [34]. However, above 15 mm to 40 mm, tensile strength remained approximately constant [34]. In injection-molded poly[styrene-b-(ethylene-co-butylene)-b-styrene) (SEBS)/HFRP composite with 30 wt.% HF), increasing nominal fiber length from 1.10 mm to 4.19 mm (from 0.57 mm to 1.03 mm after injection molding) significantly raised tensile strength from ~33 to ~39 MPa, and tensile modulus from ~2.3 to ~2.9 GPa [37]. Sajin et al. found for a compression molded alkali-treated JF-reinforced isopthalic polyester composite (JFRP), 20 mm fiber length gave the highest tensile strength and modulus, flexural strength and modulus, and impact strength. The 20 mm fiber length samples had higher mechanical properties than either the 5, 10, 15, or 25 mm samples [39]. In a compression-molded sisal fiber-reinforced PP (SFRP) composite with 40 wt.% SF, increasing chopped SF length from ~5 mm to ~25 mm (from ~3 mm to ~17 mm after compounding) resulted in increase in tensile strength from ~36 to ~43 MPa, flexural strength from ~41 to ~62 MPa, flexural modulus from 1.4 to ~2.9 GPa, and impact strength from 3.25 to 4.09 kJm^−2^ [40]. Moreover, for a biodegradable composite fabricated from agave leaves and epoxy (AFRP), longer AF lengths of 60 mm were found to give better tensile and flexural properties than those that were 10 mm or chopped [41]. Hence, in 2-phase fiber-polymer systems, mechanical properties typically decrease with a decrease in fiber length.

However, counter to the results stated in the literature for numerous 2-phase fiber/polymer composite systems, in the 3-phase SGFRP-BMC, several mechanical properties have been increased by decreasing fiber length below that of commercially used samples [7,8,36]. For a widely-used compression-molded styrene butadiene SGFRP-BMC with 11 wt.% SGF and 55 wt.% CaCO_3_ filler, it was found that shortening SGF length from nominal (commercial) 6.4 mm to submillimeter 0.44 mm raised impact strength 25% at the typically weak center of the mother panel [36]. Moreover, for an injection-molded styrene-butadiene SGFRP-BMC with 20 wt.% SGF, and 47.1 wt.% CaCO_3_ filler, shortening SGFs from 6.4 mm to 0.44 mm increased tensile modulus 5 to 25% [8]. Ultimate tensile strength (UTS) and its strain were also boosted by ~60 and ~40%, respectively [7]. This was unusual, since there is typically a tradeoff: as UTS is enhanced, its strain is typically decreased [16]. Other mechanical tests, such as flexural and fatigue, would provide a more thorough characterization, but are beyond the scope of this study.

**Figure 1:**
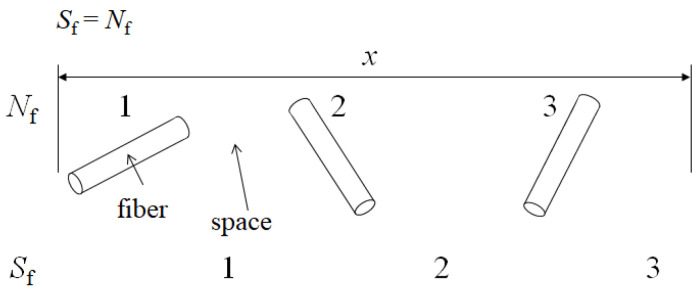
Fiber spacing model adapted from Faudree et al. (2014) [8].

In the above cases, SGFs were shortened by 30 min in an extended mix of the paste in a sigma-blade mixer prior to molding without a change in molding equipment. Enhancements by shortening fibers has been described with a “fiber spacing” model [8] depicted in Figure 1. For the simple case when fibers are separated by spaces in a given length, *x*, number of fibers, *N*_f_ will be equivalent to number of spaces, *S*_f_, as shown in Equation (1):

*S*_f_ = *N*_f_ (1)

This would be independent of fiber orientation, *θ*, with respect to *x*. And apply to the random fiber orientation of the SGFRP-BMC panel center. Moreover, since both *N*_f_ and *S*_f_ are dimensionless quantities, they can be put into any dimensional coordinate system; hence, *S*_f_ is given in three dimensions as “fiber spacing density” (mm^−3^). 

Enhancements of mechanical properties were attributed to the increase in *S*_f_ as mean fiber length, *l*_f_ (mm) is shortened, acting to increase the micro-compressive stress sites of the matrix on the SGFs by a difference in coefficient of thermal expansion (CTE) during cooling down and shrinking [8]. *S*_f_ is related to *l*_f_ by the following Equation [8]:

*S*_f_ = *ρ*_f_ = *V*_f_/(π*r*^2^*l*_f_) (2)

where *V*_f_ and *r* are SGF volume fraction and mean fiber radius (0.007 mm). It follows that the CTE of cured polyester resin matrix is 55 to 100 × 10^−6^/K [42], an order of magnitude above E-glass fibers at 5.4 × 10^−6^/K [43]. In concert with this, since the SGFRP-BMC is a three-phase fiber-filler-polymer system, the filled matrix behaves as a polymer-filler subsystem in the narrow spacing between fibers that efficiently allows for an increase in mechanical properties by decreasing the fiber length. During cooling down and shrinking, the polymer compresses onto the hard CaCO_3_ filler with a high surface area of the 1 to 7 μm CaCO_3_ particles, whose listed CTE is ~4.6 × 10^−6^/K [44]. As *S*_f_ is increased by reducing SGF length, micro-compressive stress sites are increased, raising the mechanical properties. 

Note that in the SGFRP-BMC under study, *V*_f_ of hard components of SGFs plus CaCO_3_ filler is quite high at 46%, with the polymer mixture at 54%. Small particles that are highly dispersed and in close proximity are excellent for maximizing thermal residual stresses [45] for strengthening the BMC composites. Another point here is in the case of boosting the ultimate tensile stress and strain; the increase in *S*_f_ also acts to halt cracks before propagating above the critical length for the SGFRP-BMC to take on more of the load [7]. Therefore, for the 3-phase SGFRP-BMC, shortening the fiber length has been found to increase mechanical properties. 

Other studies on shorter fiber lengths increasing mechanical properties are nearly non-existent. However, Senthilrajan et al. found, for a jute reinforced polyester (JFRP), at JF content of 25 wt.%, 5 mm fiber length samples had higher flexural strength and modulus, and a specific strength and modulus than those with longer lengths of 10, 15, 20, or 25 mm [38]. Their scanning electron microscope (SEM) analysis attributed the strengthening to stronger JF/Matrix bonding [38]. 

As mentioned previously, the CaCO_3_ filler particles of the SGFRP-BMC appear to act to raise tensile strength and its strain, along with tensile modulus, and impact strength when SGF length is reduced [7,8]. Metal matrix composites (MMC) and cemented carbide composites, as well as ceramics, are reported to exhibit this strengthening mechanism, in that reduced particle size increases mechanical properties [46,47,48]. Improvements were attributed to increased residual compressive stress sites by the CTE mismatch between the particles and the matrix. In the SGFRP-BMC, CaCO_3_ filler particles were measured using SEM to be in about the same size range, <1 to 7 μm [8], as that for ceramics at <1 μm to several microns [46,47,48]. 

Concerning the influence of filler on BMCs, little research has been performed in the literature. An important study for sustainability for hard-to-recycle BMCs was conducted by Matykiewicz et al., who crushed waste thermosetting BMC, using it as reinforcement in an epoxy composite [2]. Lautenschlägera et al. investigated the effects of filler for 25 vol.% jute chopped fiber-reinforced BMCs and needle-punched non-woven SMCs [14]. They showed that CaCO_3_ filler was found to produce higher tensile strength and Young’s modulus than that of paltry kaolin. In contrast, they found flexural strength had high scatter for either filler, due to inhomogeneous fiber distribution of fiber-rich and -sparse volumes [14]. Investigations on the effect of percent filler is beyond the scope of this study.

In injection-molded SGFRP-BMC, the mean SGF length of 30 min extended mix samples was measured using SEM to be sub-millimeter at 0.44 mm (std. dev. = ±0.203 mm) [1,7]. Thus, it is assumed, for the compression molded samples, that the 30 min mixing reduces SGF length to about 0.44 mm. With standard deviation of ±0.203 mm, SGF length difference between the 30 min extended mix compression-molded samples and commercial 6.4 mm samples is considered enough to obtain reliable results [7,8]. Notably, the sub-millimeter length is below that of the estimated *l*_c_ of ~1.0 mm reported for 2-phase FRPs [29,30], and *l*_c_ would depend on adhesion strength at the fiber/matrix interface. The extended mixing of 30 min is reported to have insignificant effect on the CaCO_3_ filler particles (<1 to ~7 μm) [2].

To boost impact strength further, homogeneous low-voltage electron beam (HLEBI) treatment to finished specimens with the shortened 0.44 mm SGFs will be employed. This is because, previously, 0.86 MGy HLEBI treatment to commercial 6.4 mm samples was found to increase impact values from 5 to 25% [49]. SEM showed that 0.86 MGy HLEBI resulted in much more polymer/CaCO_3_ matrix adhering to the SGFs than that of untreated, which were left virtually bare. An electron spin resonance (ESR) analysis of the samples showed HLEBI generated a strong peak (inflection point at 323.0 mT) signifying the presence of dangling bonds (DBs). The DBs are lone pair electrons in the outer orbital shells that exhibit repulsive forces acting to increase internal micro-compressive stress sites, and strengthen the SGF/Matrix interface [49]. This allows for energy dispersion in the form of higher numbers of cracks generated in the SGFRP-BMC samples to take on higher energy impacts [49]. An examination of tested samples showed that the fracture mechanism transitioned at *a*_uc_ of 5.4 to 6.7 kJm^−2^ from clean to smaller secondary micro-crack proliferation, sometimes including bends near the main crack. Fracture surface area was observed to increase with increasing impact strength. The conversion was independent of HLEBI-treated or untreated conditions [49]. 

HLEBI has a positive track record of enhancing GFRPs with long fibers, GFs themselves, silica glass, and several types of polymers [49,50]. HLEBI is also reported to strengthen the fiber/matrix interface with electronic charge generation and the formation of DBs [49].

**Figure 2:**
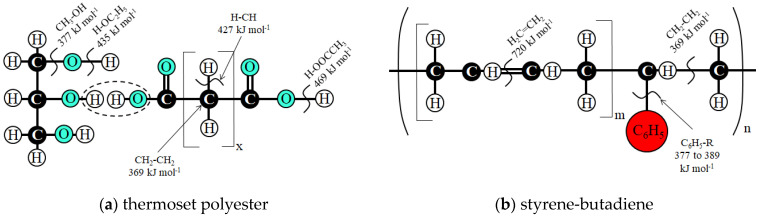
Depiction of HLEBI-activated: (**a**) thermoset polyester, and (**b**) styrene-butadiene co-polymer, respectively, adapted from Faudree et al. (2012) [49], indicating approximate bond dissociation energies (BDE) [51,52] and DBs.

HLEBI typically severs bonds with the lowest BDE (Figure 2a,b) for polymeric components in the SGFRP-BMC. DBs are formed at the terminated atoms with low BDE [49] in methylene groups (CH_2_–CH_2_: 369 kJmol^−1^), hydroxyl groups (CH_3_–OH: 377 kJmol^−1^), and phenyl groups (C_6_H_5_–R: <377~389 kJmol^−1^ where R– is CH_3_– or C_2_H_5_–). Free-radical hydrogens have higher BDE (H–CH: 427 kJmol^−1^, H–OC_2_H_5_: 435 kJmol^−1^, and H–OOCCH_3_: 469 kJmol^−1^). Alkene groups H_2_C=CH_2_ have the highest BDE at 720 kJmol^−1^ [51,52].

The action of HLEBI results in atoms being spaced further apart by DB generation, creating compressive stress sites by repulsion of the generated lone-pair electrons. For silica glass, for instance, in the radial distribution function (RDF), the normalized coordination number (*N*_D_/*N*_O_) is reduced, while the normalized mean atomic distance, (*r*_D_/*r*_O_) is increased [49]. This results in nano-scale volume expansion that can act to increase mechanical properties. 

As mentioned earlier, we strengthen the weak center of the SGFRP-BMC panel by shortening SGFs followed by HLEBI. This is because separate studies of shortening SGFs from commercial 6.4 mm to 0.44 mm [36]; and applying 0.86 MGy HLEBI to finished 6.4 mm samples [49] were found to successfully raise impact energy of the weak center of the SGFRP-BMC mother panel, 25%, and 5 to 25%, respectively. Hence, it seems possible that the combination can additively boost impact strength by 50%. Therefore, we chose the independent parameters of (1) 6.4 mm SGFs, (2) 0.44 mm, and (3) 0.44 mm + 0.86 HLEBI to illustrate the enhancements. The 6.4 mm is chosen since it is widely used commercially, while the 0.44 mm was found to yield significant increase in mechanical properties [7,36]. In addition, the 0.86 MGy HLEBI dose was chosen since preliminary testing showed that it produced the highest impact values out of a wide range of dose levels [49]. Therefore, the goal of this study is to apply a double-step process of (1) shortening the nominal 6.4 mm fiber length formulation to 0.44 mm by 30 min extended mixing without change in molding equipment, followed by (2) applying a 0.86 MGy dose of HLEBI to both sides of the SFGRP-BMC finished samples with no chemicals or additives to boost impact values at the weak center of the mother panel. 

## 2. Materials and Methods 

### 2.1. Preparation of SGFRP-BMC Samples

**Figure 3:**
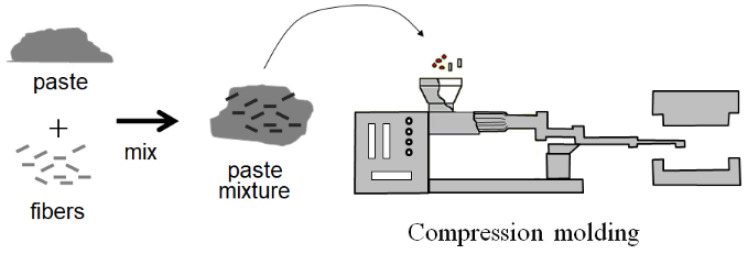
Schematic of compression molding process.

Compression-molded SGFRP-BMC panels were provided by Premix, Inc., (now Citadel Plastics) North Kingsville, OH, USA, with components listed in Table 1. A strong coupling agent was used on the SGFs, but its components are proprietary. Processing parameters, panel geometry, and volume fractions are listed in Table 2. A schematic of the compression molding process is illustrated in Figure 3, where SGFs are mixed into a paste prior to injection molding. The test matrix is composed of three data sets listed in Table 3 designated as “6.4 mm”, “0.44 mm”, and “0.44 mm + HLEBI” samples. The “6.4 mm” data set is the commercial formulation SGFRP-BMC with nominal 6.4 mm SGFs which underwent 20 min mixing of the paste with a double-arm sigma blade Banbury mixer before injection molding. The “0.44 mm” are those with an extended mix of 30 min, equaling 50 min mixing total. Mean SGF length was found to be 0.44 mm [36]. The 6.4 mm and 0.44 mm samples were not treated with HLEBI. A third data set, the “0.44 mm + HLEBI”, was treated with 0.86 MGy HLEBI on both sides of the finished samples. 

**Figure 4:**
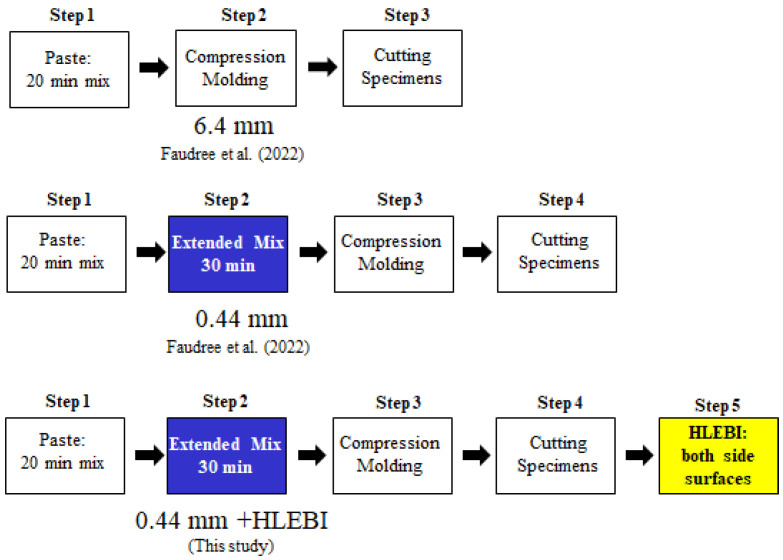
Steps of SGFRP-BMC fabrication for.4 mm; 0.44 mm (Faudree et al. (2022)) [36]; and 0.44 mm + HLEBI samples, respectively.

Figure 4 shows the fabrication steps for the three data sets, (a) 6.4 mm, (b) 0.44 mm, and (c) 0.44 mm + HLEBI. The commercial 6.4 mm and 0.44 mm samples were fabricated in three and four steps, respectively [36]. Figure 4 shows that the 0.44 mm + HLEBI samples were fabricated in five steps. Step 1 is mixing the paste for 20 min. Step 2 is 30 min of extended mixing. Step 3 is compression molding the paste into panels. Step 4 is cutting the samples. Finally, Step 5 is applying 0.86 MGy HLEBI to both sides of the samples (see the HLEBI Section). 

**Figure 5:**
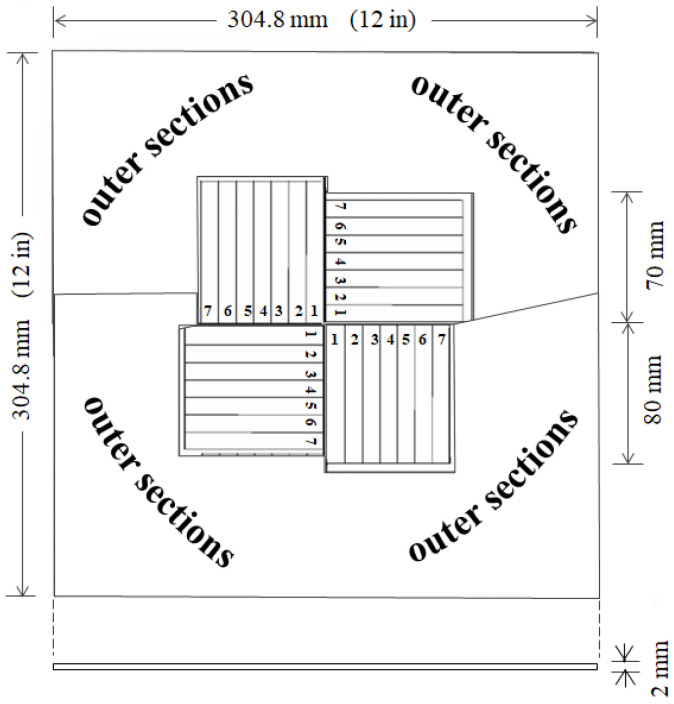
Schematic of Charpy impact sample cutting from the weak center section of a SGFRP-BMC panel. Specimen numbers indicated. (outer section specimens not shown).

Figure 5 is an illustration of the SGFRP-BMC panels, showing specimens taken from the weak center section. During compression molding of the paste charge, the direction of flow is outward, but more random just below the plunger in the center. After molding, specimens (80 × 10 × 2 mm) were cut according to ASTM D 6110-02 (2002) [53] for anisotropic panels [53]. Since this study focusses on improving impact strength at the weak panel center, samples cut from outer 3 sub-quadrants of each quadrant are not shown. Only the 4 sub-quadrants at center are shown, and are assumed to have identical flow patterns since they exhibited statistically lower impact strength than the outer sections [1,36,49]. Locations of samples cut from the panel center are indicated in Figure 5. For consistency, specimens in each sub-quadrant are numbered from 1 to 7 outward from the center. The 14-sample data sets were taken from two sub-quadrants, two each from sample numbers 1 to 7.

### 2.2. Homogeneous Low-Voltage Electron Beam Irradiation

**Figure 6:**
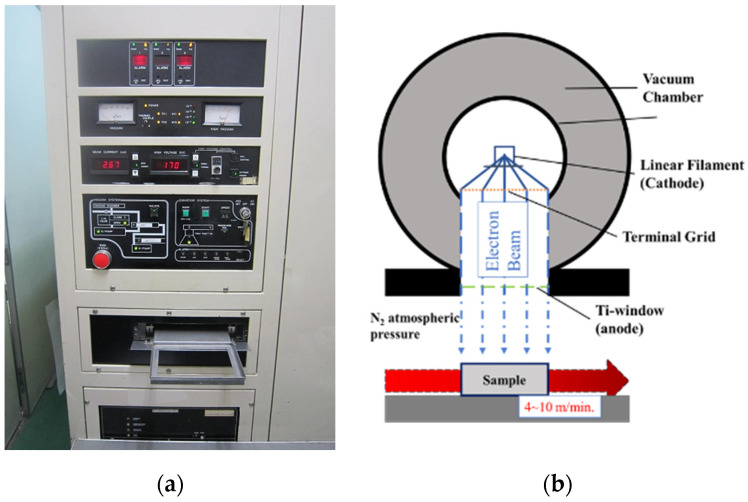
Electron-curtain processor (Iwasaki): (**a**) photo, and (**b**) schematic.

After molding and cutting, samples were treated with a HLEBI processor (Table 4) (Type LB250/15/180L, Energy Science, Inc., Woburn, MA, USA, Iwasaki Electric Group, Ltd., Tokyo, Japan), as shown in Figure 6a,b [49]. Faudree et al. (2022) gives a detailed explanation [36]. To estimate how far into sample thickness HLEBI activates, penetration depth, *D*_th_ (μm) is calculated. When *ρ* is sample density (g cm^−3^) and *V* is acceleration voltage at sample surface (kV), the *D*_th_ can be obtained [54]:

*D*_th_ = 66.7*V*^5/3^/*ρ* (3)

Individual *D*_th_ are listed in Table 5. *D*_th_ for the SGFRP-BMC is 116 μm, or about 5.8% into the thickness. Since both specimen sides are HLEBI-activated, there is a skin/core/skin sandwich structure of 0.116/1.87/0.116 mm. Impact tests were carried out 30 ± 0.5 h after HLEBI irradiations.

### 2.3. Charpy Impact Tests

**Figure 7:**
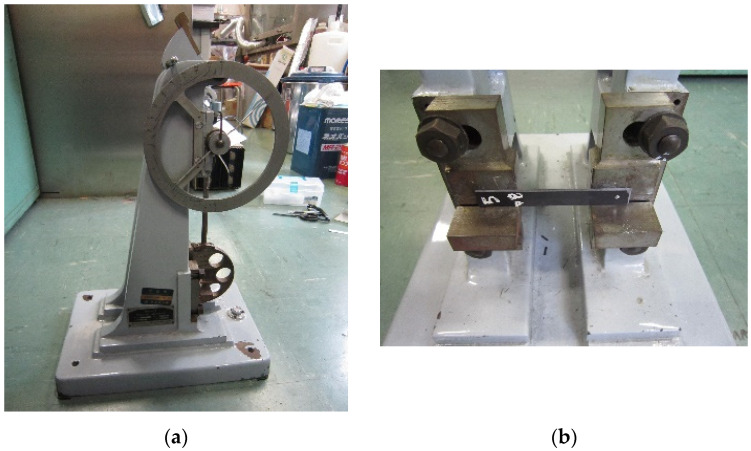
Photos of: (**a**) Charpy impact tester, and (**b**) mount with SGFRP-BMC specimen.

Charpy impact strength was measured using a drop-weight pendulum apparatus (Shimadzu Corp. No. 51735, Tokyo, Japan) [55,56] according to the JIS K 7077-1991 testing standard [55]. Figure 7a,b shows the impact tester and specimen mount. Specimen dimensions were 80 × 10 × 2 mm. Testing parameters and details can be found in Faudree et al. (2022) [36]. 

## 3. Results 

### Effect of Shortening SGFs and HLEBI on Impact Values

**Figure 8:**
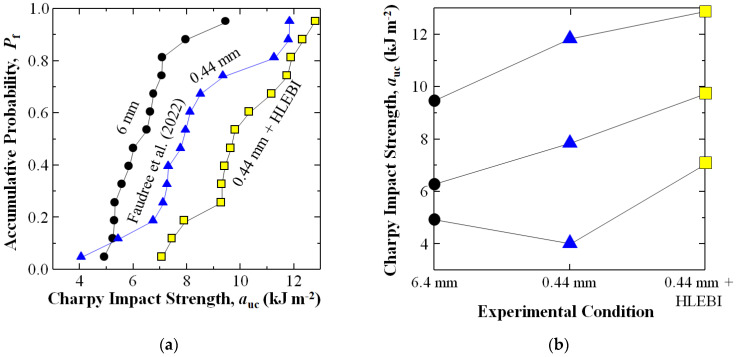
Experimental results showing: (**a**) changes in Charpy impact values *a_uc_* (kJm^−2^) for SGFRP-BMC samples of untreated 6 mm and 0.44 mm, along with 0.44mm + HLEBI (0.86 MGy), with (**b**) the *a*_uc_ from (**a**) *a*_uc_ at low-, median-, and high-*P*_f_ of 0.049, 0.500, and 0.951, respectively. The 0.44 mm data are from Faudree et al. (2022) [36].

Experimental results are shown in Figure 8a,b for the 6.4 mm (black dots), 0.44 mm (blue triangles) [36], and 0.44 mm + HLEBI (yellow squares) samples, respectively. Figure 8a shows accumulative probabilities (*P*_f_) vs. Charpy impact value (*a*_uc_) according to a general form of the median rank method [57] described in detail in Faudree et al. (2022) [36]. Figure 8a shows, for the 0.44 + HLEBI data set, our double-step process of (1) shortening fibers to 0.44 mm with 30 min extended mixing, followed by (2) HLEBI of 0.86 MGy, boosted *a*_uc_ at all *P*_f_ over that of the 6.4 mm. The 0.44 mm data set (without HLEBI, blue triangles) is from a previous study reported by Faudree et al. (2022) [36], and is shown here to illustrate that the 0.44 mm + HLEBI data set yields higher *a*_uc_ than the 0.44 mm at all *P*_f_. Although at low-*P*_f_ of 0.049, *a*_uc_ of the 0.44 mm data set was reduced (4.06 kJm^−2^) from that of the 6.4 mm (4,.92 kJm^−2^) [36], the 0.44 mm + HLEBI process showed a significant improvement to 7.06 kJm^−2^. As stated in [36], the *a*_uc_ are calculated from (*a*_uc_ = *E*/(*bt*)) with impact energy, and *E* is obtained with (*E* = *WR*[(cos*β* − cos*α*) − (cos*α*’ − cos*α*)(*α* + *β*)/(*α* − *α*’)]). The *P*_f_ are calculated from (*P*_f_ = (*I* − 0.3)/(*N*_s_ + 0.4)). 

Figure 8b shows *a*_uc_ at low-, median-, and high-*P*_f_ of 0.049, 0.500, and 0.951, respectively. Namely, the 0.44 mm + HLEBI data set shows significant increase in *a*_uc_ over that of 6.4 mm. At *P*_f_ of 0.049, 0.500, and 0.951, *a*_uc_ was increased 43%, 55%, and 35%, respectively, from 4.92 to 7.06 kJm^−2^, 6.26 to 9.72 kJm^−2^, and 9.45 to 12.80 kJm^−2^. For the 0.44 mm data set, Figure 8b shows the increases in *a*_uc_ at median- and high-*P*_f_, but a decrease at low-*P*f of 0.049 compared with those of 6.4 mm [36]. However, the 0.44 mm + HLEBI process significantly raises the *a*_uc_ over those of the 6.4 mm samples at low-*P*_f_ of 0.049, along with those at median-, and high *P*_f_. 

Table 6 provides individual *a*_uc_ for each sample of the data sets in Figure 8. 

## 4. Discussion 

### 4.1. Average Impact Strength

**Figure 9:**
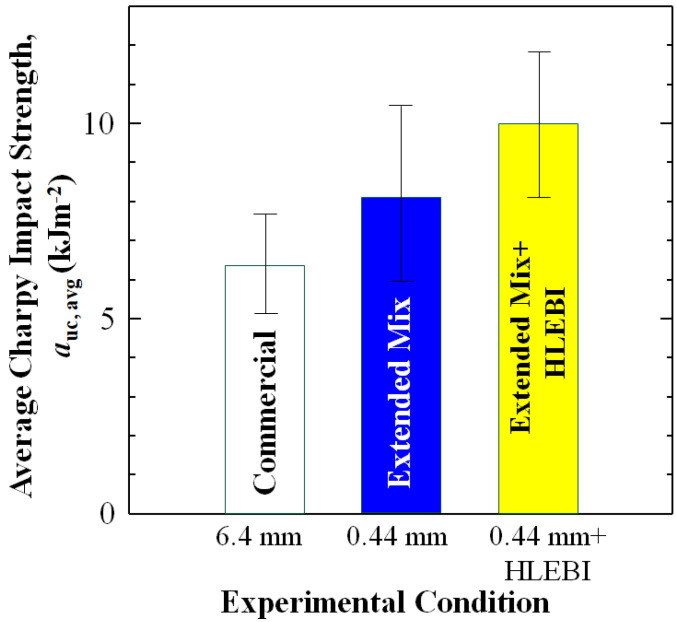
Average impact strength, *a*_uc, avg_ (kJm^−2^) with standard deviations (bars) for the three data sets: 6.4 mm, 0.44 mm, and 0.44 mm + HLEBI, respectively.

Since average values are commonly employed in strength evaluations, Figure 9 is included here showing the average *a*_uc_ (*a*_uc,avg_) with standard deviation bars. Figure 9 shows the double-step process of 0.44 mm + HLEBI significantly increased *a*_uc,avg_ 56% over that of 6.4 mm from 6.41 to 10.00 kJm^−2^. Although standard deviations were quite high at 1.24 and 1.81 kJm^−2^ for the 6.4 and 0.44 + HLEBI data sets, respectively, the lower limit of the 0.44 mm + HLEBI (8.19 kJm^−2^) was higher than the upper limit of the 6.4 mm (7.65 kJm^−2^). This provides further support that our double-step process can apparently increase the *a*_uc_ of weak center sections of the SGFRP-BMC panels. 

### 4.2. Micro-Strengthening Mechanism Using Extended Mix

**Figure 10:**
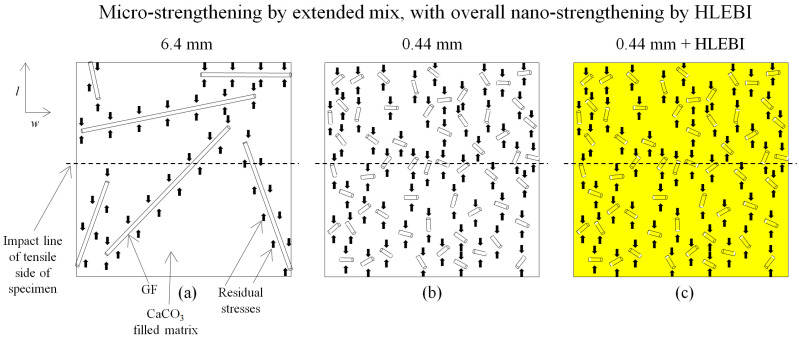
Schematic of micro-scale strengthening mechanism showing (**a**) 6.4 mm; (**b**) 0.44 mm; and (**c**) 0.44 mm + HLEBI samples, respectively. In (**c**), yellow indicates HLEBI activation. Specimen tensile sides are depicted with line of impact. Specimen length and width directions are indicated.

**Figure 11:**
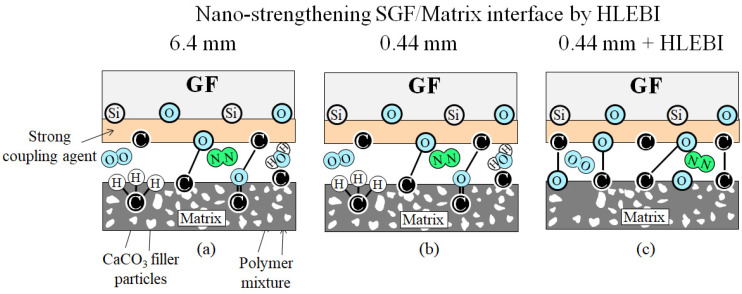
Schematic of the nano-scale SGF/Matrix interface-strengthening mechanism by HLEBI depicting an increase in strong bonds for: (**a**) 6.4 mm; (**b**) 0.44 mm; and (**c**) 0.44 mm + HLEBI samples, respectively.

Figure 10a–c illustrates the micro-strengthening mechanism by an extended mix of the SGFRP-BMC for the 6.4 mm, 0.44 mm, and 0.4 mm + HLEBI specimens. The SGFs are depicted as having a random orientation as in the panel center. Impact strength enhancements are attributed to increase in *S*_f_ and *N*_f_, as *l*_f_ is shortened, acting to increase micro-compressive stress sites of the matrix on the SGFs by a difference in CTE during cooling down and shrinking [8,36]. The composite is hardened, increasing impact resistance. Figure 11b shows an order of magnitude increase in *S*_f_ and *N*_f_ over that of 6.4 mm (a) that is exceptionally more dispersed. Here, *N*_f_ is made to approximate the actual situation where a total length of 58 of 0.44 mm SGFs equals 4 of 6.4 mm SGFs. To discuss enhancement during impact, Figure 10 is made to depict tensile side of specimen with impact area across specimen width (dotted lines). Shortening SGFs to 0.44 mm allows for a higher proportion of SGFs to cross the line, along with increased compressive stress sites (arrows), to counter tensile deformation from impact for higher *a*_uc_. On the other hand, in Figure 10a, the less dispersed 6.4 mm samples have gaps in the form of areas lacking SGFs with less residual stresses resulting in easier crack initiation and propagation in the matrix at the tensile side when impacted.

As mentioned earlier, the filled matrix behaves as a polymer-filler subsystem in the spacing between fibers allowing for an increase, but not a decrease, in mechanical properties with decreasing fiber length. To increase *a*_uc_ further, Figure 10c depicts the nano-scale strengthening of the 0.44 mm composite with HLEBI represented in yellow. 

### 4.3. Nano-Strengthening Mechanism Using HLEBI

**Figure 12:**
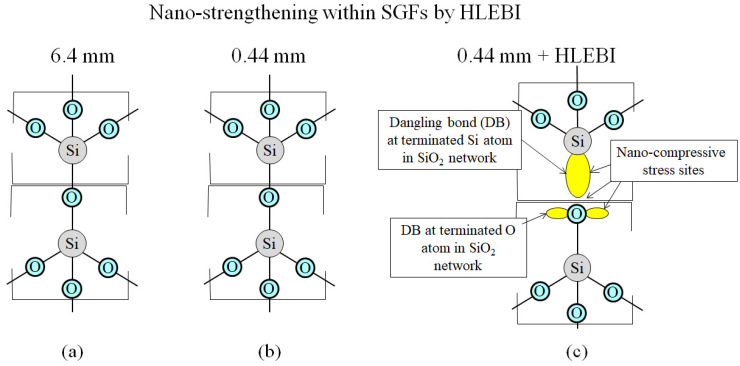
Schematic of the nano-scale strengthening mechanism within SGFs by HLEBI for: (**a**) 6.4 mm; (**b**) 0.44 mm; and (**c**) 0.44 mm + HLEBI samples, respectively.

To illustrate the nano-strengthening mechanism, action of HLEBI occurs at: (1) the SGF/Matrix interface; (2) within the SGFs; and (3) within the polymer matrix. These are explained in Figure 11 and Figure 12, and previously in Figure 2a,b. HLEBI works by severing bonds, creating DBs in the form of lone pair electrons that enhance interfacial adhesion and strengthen bulk materials. When 0.86 MGy HLEBI dose is applied to both surfaces of finished SFGRP-BMC samples at the weak panel center, *a*_uc_ can be raised. Figure 11a,b shows, for untreated 6.4 mm and 0.44 mm, that the SGF/Matrix interface has typical strong chemical bonds between the SGFs and the matrix from the coupling agent. They are apparently accompanied by weak Van der Waals forces with trace atmospheric gasses, O_2_, N_2_, and H_2_O existing at the interface. However, Figure 11c depicts that when HLEBI is applied in the 0.44 mm + HLEBI samples, additional strong bonds of C-C and C-O are apparently formed by lone electron pairs generated, i.e., DBs, raising the *a*_uc_. As mentioned earlier, DBs have been detected in 0.86 MGy HLEBI-treated 6.4 mm fiber length SGFRP-BMC as peak generation using ESR analysis in a previous study [49]. Moreover, SEM revealed the HLEBI increased matrix adhering to SGFs, with increased impact values ~5 to 25% [49]. 

Figure 12a–c illustrates the strengthening within SGFs by HLEBI. HLEBI has been reported to strengthen silica glass [49]. Figure 12a,b shows the unactivated bonds in the SGFs of 6.4 mm and 0.44 mm samples. However, Figure 12c shows HLEBI activation creates DBs in the outer shell electrons at terminated O atoms in the SiO_2_ network in the 0.44 mm + HLEBI samples. The repulsion between lone pairs creates nano-compressive stresses that strengthen SGFs themselves to assist in raising the *a*_uc_ of the SGFRP-BMC system. 

To summarize, the new double-step process of shortening SGFs using a 30 min extended mix, followed by 0.86 MGy HLEBI to finished samples, was found to increase the *a*_uc_ at the weak center of the SGFRP-BMC compression molded mother panel. This is caused by micro-strengthening by shortening fibers, as well as nano-strengthening by HLEBI. 

However, for maximum safety, carefulness is highly recommended, since this study only applies to the panel center. HLEBI to the outer sections may weaken the composite. Also, it is always imperative to test for optimum dose of HLEBI for each situation. Nevertheless, the double-step process was found to increase impact values significantly, over 50%, at the weak center of the SGFRP-BMC mother panel.

### 4.4. Environmental Sustainability and Long-Term Durability Aspects

With the sharp increase in catastrophic events and heavy degradation to Earth’s environment, environmental sustainability must be top priority in manufacture of any product. Therefore, we employ the double-step process to increase the impact strength of the SGFRP-BMC of the 30 min extended mix without a change in molding equipment, followed by HLEBI treatment without the use of any chemicals. However, for a full evaluation of the ecological footprint, life cycle assessment (LCA) is typically carried out. Energy consumption analysis of the double-step process would have to be taken into account. For the LCA to compare that with and without the double-step process would be needed. The LCA is evaluating the continuous cycle of raw materials, manufacturing, transportation, usage and selling, waste disposal, and recycling. The SGFRP-BMC is difficult to recycle, since the polymeric matrix is a thermoset that cannot be melted, for example, to separate it with filler and SGFs. Dumping SGFRP-BMC parts in a landfill is extremely hazardous for the environment and should be strictly prohibited. A highly employed remedy is to ground the waste GFRP-BMC and used as a filler [2]. Use of the filler recyclate for thermoplastic FRP would be recommended, since thermoplastics can be repeatedly melted and solidified for recyclability. As for long-term durability assessment, aging studies to check for mechanical strength reduction with time in treated SGFRP-BMC would be needed. LCA and aging studies are beyond the scope of this study, but should be considered for future research. 

### 4.5. Economic Analysis, Scale-Up, and Feasibility Studies

Economic analysis, scale-up, and feasibility information are proprietary and beyond the scope of this study. 

## 5. Conclusions

In order to maximize the safety of outdoor articles, airplanes, and space vehicles by protecting against the impact of airborne debris from increasing winds due to climate change, or from bird strikes or micrometeoroids, it is imperative for composite materials to have high impact resistance. In a 3-phase compression-molded short glass fiber polyester bulk-molded compound (SGFRP-BMC) with 55% wt. CaCO_3_ filler and 11% wt. SGF, the center of the mother panel has lower impact strength than the outer sections, with solidification texture angles and SGF orientations being random from 0 to 90 degrees.

1. Therefore, a new double-step process of: (1) shortening the nominal 6.4 mm fiber length formulation to submilllimeter 0.44 mm by 30 min extended mixing without change in molding equipment, followed by (2) applying 0.86 MGy dose of homogeneous low-voltage electron beam irradiation (HLEBI) to both sides of the finished samples, requiring no chemicals or additives, which is shown to increase Charpy impact value (*a*_uc_) over 50% from 6.26 to 9.59 kJm^−2^ at a median-accumulative probability of fracture, *P*_f_ = 0.500.
2. Shortening the SGFs by the extended mix method to submillimeter creates a higher number of thermal micro-compressive stress sites between SGF and the matrix to increase impact strength. This is performed by a mismatch of the coefficient of thermal expansion (CTE) between the matrix and fibers acting in the increased fiber spacing density while the composite is undergoing cool-down and shrinkage. In concert with this, since the SGFRP-BMC is a 3-phase fiber-filler-polymer system, the filled matrix behaves as a polymer-filler subsystem in the narrow spacing between fibers that efficiently allows for the increase in mechanical properties by decreasing the fiber length.
3. To boost impact strength further, HLEBI additionally provides nano-compressive stresses in the matrix by generating a dangling bond, which acts as repulsive force site between the outer-shell lone-pair electrons. This, along with increasing SGF/matrix adhesion, occurs with the optimum dose of HLEBI. During impact, a higher degree of internal cracking apparently occurs, raising the impact strength of SFGFRP-BMC samples.

## Figures and Tables

**Table 1:** Weight percents of SGFRP-BMC components.

COMPONENT	Wt.%
Propylene glycol maleate polyester	13.75
Styrene butadiene copolymer	12.75
Commercial E-glass fibers	11
CaCO_3_ filler	55
Aluminum silicate filler	3
Magnesium hydroxide	0.5
Proprietary initiators and inhibitors	4

**Table 2:** Processing parameters.

PARAMETER	Condition
Mold pressure	5.5 to 6.9 MPa (800 to 1000 psi)
Temperature	422 K (149 °C)
Cure time	2 min
Mold type	Matched metal die compression mold
Panel size	304.8 × 304.8 mm (12 × 12 in)
Panel thickness	2 mm
*V*_f_ (SGF)	0.080
*V*_f_ (CaCO_3_)	0.377
*V*_f_ (remaining polymer mixture)	0.543

**Table 3:** Data sets of the SGFRP-BMC.

Experimental Condition	6.4 mm Untreated	0.44 mm Untreated	0.44 mm + HLEBI
Number of Specimens	14	14	14

**Table 4:** HLEBI parameters.

Acceleration voltage	170 kV
Irradiating current	2.68 mA
Irradiation environment	N2 gas atmosphere
Residual O_2_ conc.	<300 ppm
Conveyor speed	10 m min^−1^
EB dose/sweep	0.0432 MGy
Sweep time (one way)	0.20 s
Gap interval bet. sweeps	20 s
EB yield calc.	FWT Nylon dosimeter

**Table 5:** Densities and the calculated *D*_th_ for the SGFRP-BMC (bold type) and its individual components [54].

Component	Density, *ρ* (g cm^−3^)	*D*_th_ (μm)
Polymer Mixture	1.200	185
Matrix Compos.	1.847	120
**SGFRP-BMC**	**1.917**	**11** **6**
SGF	2.620	84.5
CaCO_3_ Filler	2.800	79.1

**Table 6:** Charpy impact values, *a*_uc_ (kJm^−2^), of individual specimens along with their accumulative probabilities, *P*_f_.

*P*_f_	6.4 mm Untreated	0.44 mm Untreated	0.44 mm+ HLEBI
0.049	4.92	4.06	7.06
0.118	5.24	5.44	7.45
0.188	5.29	6.74	7.91
0.257	5.32	7.12	9.28
0.326	5.57	7.26	9.30
0.396	5.84	7.31	9.41
0.465	6.01	7.76	9.64
0.535	6.51	7.96	9.80
0.604	6.64	8.12	10.33
0.674	6.76	8.51	11.16
0.743	7.06	9.35	11.75
0.813	7.09	11.26	11.89
0.882	7.97	11.79	12.33
0.951	9.45	11.84	12.80

## Data Availability

Experimental data for this manuscript can be obtained upon request by contacting M.C. Faudree of Tokyo City University (faudree@tcu.ac.jp) or Yoshitake Nishi of Tokai University (west@tsc.u-tokai.ac.jp).

## Abbreviations

The following abbreviations are used in this manuscript.

SGFRP-BMC	short glass fiber reinforced polymer bulk molding compound
SGF	short glass fiber
CTE	coefficient of thermal expansion
HLEBI	homogeneous low-voltage electron beam irradiation
DB	dangling bond
FRP	fiber-reinforced polymer
**Symbols**	
*a*_uc_	Charpy impact strength (kJm^−2^)
*S*_f_	fiber spacing density (mm^−3^)
*N*_f_	fiber number density (mm^−3^)
*V*_f_	volume fraction
*r*	fiber radius (mm)
*l*_f_	fiber length (mm)
BDE	bond dissociation energy (kJ mol^−1^)
*D*	HLEBI irradiation dose (MGy)
*I*	HLEBI irradiation current (mA)
*S*	HLEBI conveyor speed (mmin^−1^)
*N*	HLEBI number of irradiations
*D*_th_	HLEBI penetration depth (µm)
*V*	HLEBI acceleration voltage (V)
*ρ*	sample density (g mm^−3^)
*E*	impact fracture energy (kJ)
*W*	impact hammer mass (kg)
*R*	length of hammer weight point from pivot center (m)
*β*	maximum angle after impact (Rad)
*α*	start angle before impact (Rad)
*α*’	maximum angle of a blank test (Rad)
*b*	specimen width (mm)
*t*	specimen thickness (mm)
*P*_f_	accumulative probability
*I*_R_	ascending strength rank
*N*_s_	total number of samples in a data set
